# Supplementary material for: Chemical inhibitors of the conserved bacterial transcriptional regulator DksA1 suppressed quorum sensing-mediated virulence of Pseudomonas aeruginosa
Source: J Biol Chem. 2021 Mar 21;296:100576. doi: 10.1016/j.jbc.2021.100576 (PMC8081920; doi:10.1016/j.jbc.2021.100576)
Supplement: Supplemental Figures S1–S5 and Tables S1–S2 [file mmc1.pdf]

Chemical Inhibitors of DksA1, a Conserved Bacterial Transcriptional Regulator, Suppressed Quorum Sensing-Mediated Virulence in *Pseudomonas aeruginosa*

Running title: Dkstatin suppressed virulence in *Pseudomonas aeruginosa*

Kyung Bae Min[1,2], Wontae Hwang[1,2], Kang-Mu Lee[1], June Beom Kim[1,2] and Sang Sun Yoon[1,2,3#]

[1]Department of Microbiology and Immunology, Yonsei University College of Medicine, Seoul, Republic of Korea, [2]Brain Korea 21 PLUS Project for Medical Sciences, Republic of Korea, [3]Institute for Immunology and Immunological Diseases, Yonsei University College of Medicine, Seoul, Republic of Korea

[#]Corresponding author
Sang Sun Yoon, Ph.D.
Department of Microbiology
Yonsei University College of Medicine
250 Seongsanno, Seodaemun-gu
Seoul, 120-752, Korea
Tel: +82-2-2228-1824
Fax: +82-2-392-7088
E-mail: sangsun_yoon@yuhs.ac

Supplementary Materials;

S1. Screening scheme for Dkstatin compound.

S2. Measurements of relative elastase activity and *rpsB* expression under four Dkstatin candidates.

S3. HSL-autoinducer complementation under Dkstatin-1 and Dkstatin-2 supplemented condition.

S4. Transcriptome analysis produced in PAO1 and PAO1 treated with Dkstatin-1 or Dkstatin-2.

S5. Fig. S5. Categorization of differentially expressed genes based on gene ontology (GO) terms.

Table S1. Bacterial strains and genetic materials used in this study.

Table S2. Primer sequences used in this work.

Table S1. Bacterial strains and genetic materials used in this study.

Bacterial strain and plasmid	Genotype or description	Reference
***E. coli* strains**		
DH5αλ*pir*	*fhuA2 lac(del)U169 phoA glnV44 Φ80' lacZ(del)M15 gyrA96 recA1 relA1 endA1 thi-1 hsdR17 λpir*	Lab collection
SM10λ*pir*	*thi thr leu tonA lacY supE recA*::RP4-2-Tc::Mu Km^r λ*pir*	Lab collection
***P. aeruginosa* strain**		
PAO1	Wild type, laboratory strain of *P. aeruginosa*	Lab collection
Δ*dksA1*	PAO1 with *dksA1* gene deletion	This study
PAO1, pRpsB::*lacZ*	PAO1 harboring a chromosomal integration of *rpsB* promoter region fused with *lacZ* gene	This study
Δ*dksA1*, pRpsB::*lacZ*	Δ*dksA1* harboring a chromosomal integration of *rpsB* promoter region fused with *lacZ* gene	This study
**Others**		
Chromobaterium violaceum CV026	Biosensor for C4-HSL	Lab collection
**Plasmids**		
pBAD24F	Amp^r cloning vector containing FLAG sequence downstream of multiple cloning site	This study
pCVD442	Amp^r suicide vector containing *sacB* for screening recombinant	(35)
pKDT17	Amp^r pTS400 plasmid containing *lasR* and *lasB* transcriptionally fused with *lacZ*	(44)
pUC18-mini-Tn7t-Gm-*lacZ*	Amp^r, Gm^r, site specific chromosomal insertion plasmid harboring promoter-less *lacZ*	(30)
pTNS2	Helper plasmid containing site specific recombinase for chromosomal insertion	(30)
pRBTn7t	pUC18-mini-Tn7t-Gm-*lacZ* plasmid harboring promoter region of *rpsB* gene	This study
pBAD24F-DksA1	Assembled pBAD24F plasmid containing *dksA1*-FLAG fusion fragment	This study
pCVD442-DksA1FLAG	Assembled pCVD442 plasmid containing *dksA1*-FLAG -downstream fragment	This study

Table S2. Primer sequences used in this work.

Primers	Sequence (5' to 3')	Description
***rpsB* reporter**		
pRpsB_F	GTACTAGTTGGCAGACCA CATGGTTGA	Promoter region of *rpsB* forward primer containing speI restriction enzyme site
pRpsB_R	ATCCCTAGGCATGATAGT CCTCGATAAG	Promoter region of *rpsB* reverse primer containing avrII restriction enzyme site
mini-Tn7t-seqF	GCTTTTGAAGCTAATTCG ATCA	Forward sequencing primer covering multiple cloning site of puc18-mini-Tn7t-Gm-l*acZ*
mini-Tn7t-seqR	TCGGGATCGCTAGTTAGT TA	Reverse sequencing primer covering multiple cloning site of puc18-mini-Tn7t-Gm-l*acZ*
**FLAG Tagging**		
pBAD24F_fwd	GACTACAAGGACCACGA C	Forward primer to amplify pBAD24F
pBAD24F_rev	AAGCTTGCATGCCTGCAG	Reverse primer to amplify pBAD24F
*dksA1*_fwd	ACCTGCAGGCATGCAAGC TTATGTCCACCAAAGCAA AACAAC	*dksA1* forward primer containing overlap region to FLAG site in pBAD24F plasmid
*dksA1*_rev	CCGTCGTGGTCCTTGTAG TCGGAGCCGAGTTGCTTC TC	*dksA1* reverse primer containing overlap region to FLAG site in pBAD24F plasmid
pBAD24FseqF	CTACTGTTTCTCCATACCG	Forward sequencing primer for FLAG-*dksA1* gene fragment in pBAD24F plasmid
pBAD24FseqR	TGCCGCCAGGCAAATTCG	Reverse sequencing primer for FLAG-*dksA1* gene fragment in pBAD24F plasmid
pCVD442_fwd	GCATGCGGTACCTCTAGA GAAG	Forward primer for amplifying pCVD442
pCVD442_rev	GAGCTCTCCCGGGAATTC	Reverse primer for amplifying pCVD442
*dksA1*FLAG_fwd	TGGAATTCCCGGGAGAGC TCATGTCCACCAAAGCAA AAC	*dksA1*-FLAG fragment forward primer containing overlap region to pCVD442
*dksA1*FLAG_rev	GTCCGGCTCATCACTTGT CGTCGTCGTC	*dksA1*-FLAG fragment reverse primer containing overlap region to 500bp downstream of *dksA1* ORF
*dksA1*downstream _fwd	CGACAAGTGATGAGCCG GACCGACGGGG	500 bp downstream of *dksA1* ORF forward primer containing overlap region to *dksA1*-FLAG fragment
*dksA1*downstream _rev	CTTCTAGAGGTACCGCAT GCCGCGGTCGACGAAGC GATATTCC	500 bp downstream of *dksA1* forward primer containing overlap region to pCVD442
P1*dksA1*FLAGse qF	CTGATCAAGAAGATCGAC GAG	Forward primer for sequencing FLAG-downstream of *dksA1* part
P1*dksA1*FLAGse qR	ACCGAAGTGCAGGTAGC CGC	Reverse primer for sequencing *dksA1*-FLAG part
P2*dksA1*FLAGse qF	CGCGACCCTCTGCATCGA CT	Forward primer for sequencing FLAG-downstream of *dksA1* part
P2*dksA1*FLAGse qR	GAACGGCGGAAACGAAA CGG	Reverse primer for sequencing *dksA1*-FLAG part
pCVD442seqF	GACACAGGAACACTTAA CGGC	Forward primer for sequencing entire *dksA1*-FLAG-downstream fragment
*dksA1*DownseqR	GGTCGACGAAGCGATATT CCA	Reverse primer for sequencing entire *dksA1*-FLAG-downstream fragment

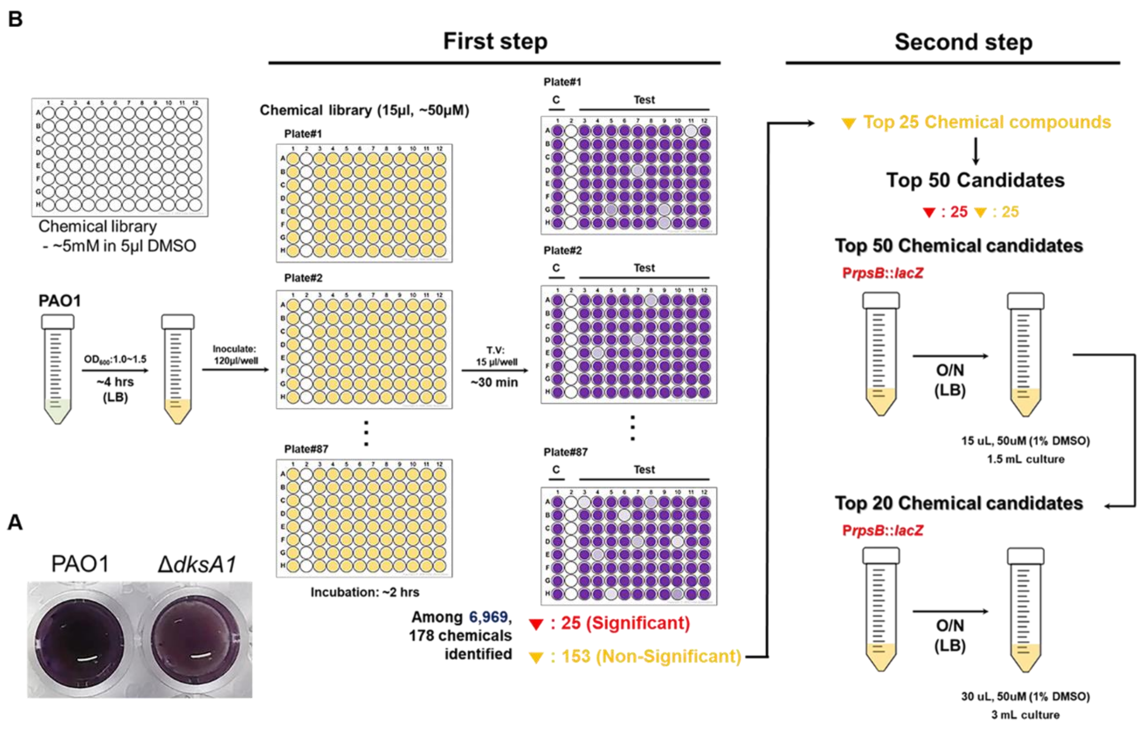

Fig. S1. Screening scheme for Dkstatin compound. (A) Visual comparison of formazan production. Reduction of formazan production in Δ*dksA1* mutant was observed. **(B)** Demonstration of screening procedure for Dkstatin compound. In the first step, the OD600 value of *P. aeruginosa* incubated in LB for 4 h was adjusted to 1.0, and it was inoculated into chemical library plates. 15 μl of chemical compounds in stock chemical libraries were distributed into new plates to at a concentration of 50 μM. Exposure of chemical compound to PAO1 was performed for 2 h at 37°C. 15 μl of 0.5 mg/ml thiazolyl blue tetrazolium bromide was inoculated into test plates to observe (purple colored) formazan production. In the second step in the screening, *rpsB* expression (represented as β-galactosidase activity) and elastase activity were measured to select further potent chemical candidates.

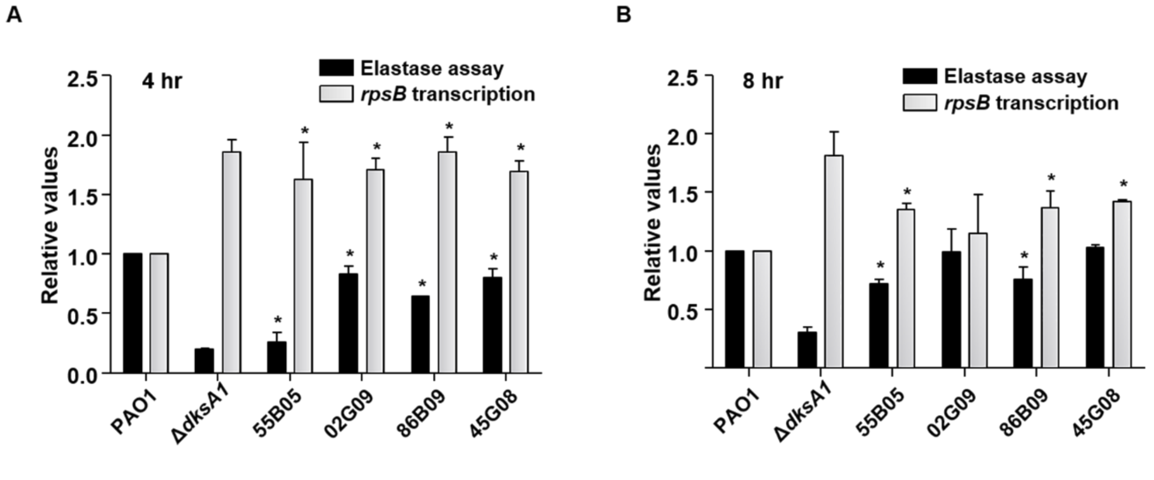

Fig. S2. Measurements of relative elastase activity and *rpsB* expression under four Dkstatin candidates. (A) Comparisons of relative elastase activity and *rpsB* expression from PAO1 P*rpsB*::*lacZ* and PAO1Δ*dksA1* P*rpsB*::*lacZ* strains incubated for 4 h in LB. Relative levels of *rpsB* expression were represented as β-galactosidase activity. 50 μM of 4 Dkstatin candidate compounds (labeled as 55B05, 02G09, 86B09 and 45G08) were supplemented in LB. The values of mean ± S.D. are presented (n=2). **(B)** Relative levels of elastase activity and *rpsB* expression of P*rpsB*::*lacZ* and PAO1Δ*dksA1* P*rpsB*::*lacZ* strains incubated for 8 h in LB. Relative levels of *rpsB* expression were represented as β-galactosidase activity. 50 μM of 4 Dkstatin candidate compounds were supplemented in LB. The values of mean ± S.D. are presented (n=2).

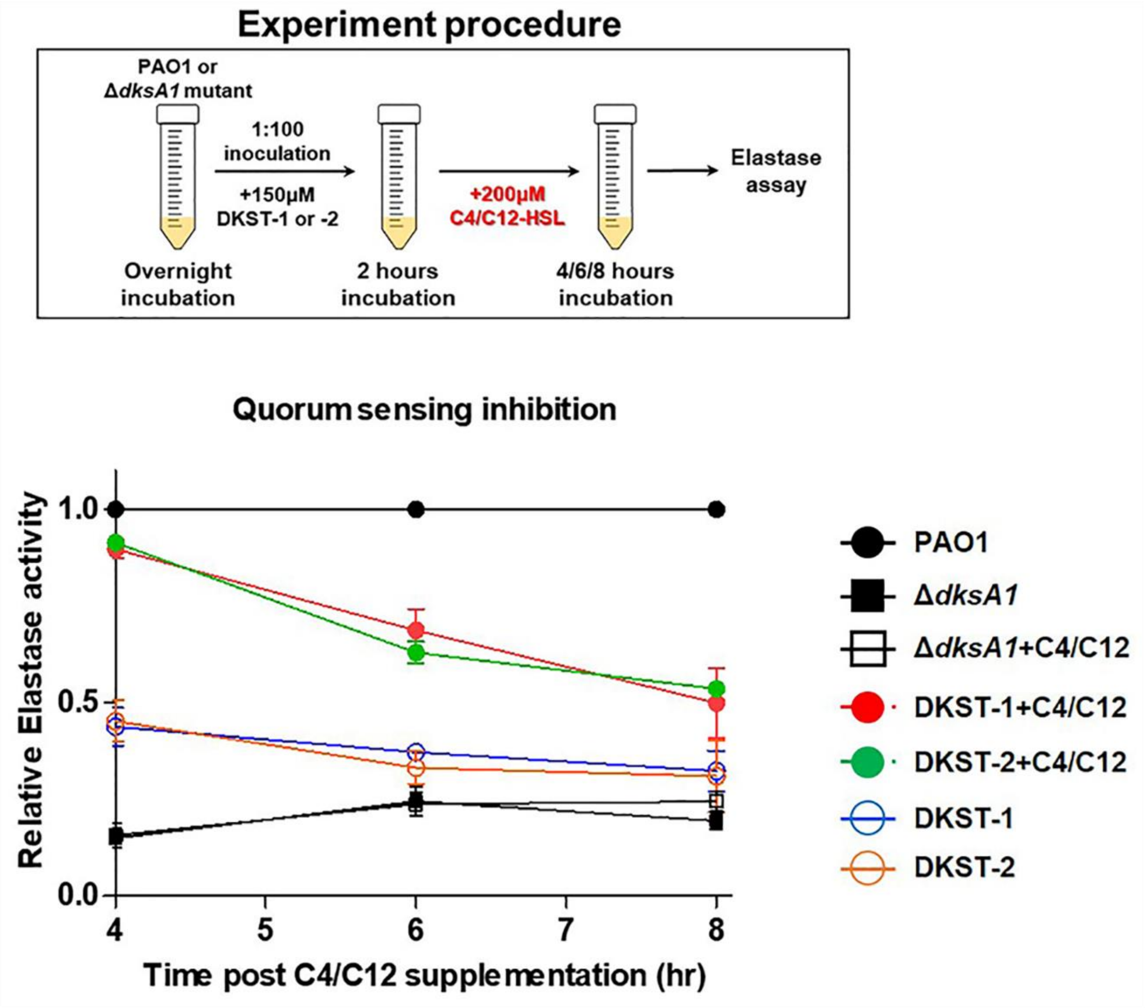

Fig. S3. HSL-autoinducer complementation under Dkstatin-1 and Dkstatin-2 supplemented condition. Demonstration of combined HSL-autoinducer (C4/C12-HSL) complementation experiment procedure (Upper). 200 μM of C4/C12-HSL complementation in PAO1 and Δ*dksA1* mutant was conducted for 8 h in LB medium with supplementation of 150 μM of Dkstatin-1 (DKST-1) or Dkstatin-2 (DKST-2). Elastase activities of PAO1 and Δ*dksA1* mutant were measured at each time point after addition of C4/C12-HSL (4, 6, and 8 h). Elastase production in PAO1 treated with 150 μM of DKST-1 (○, blue line) and DKST-2 (○, orange line) was constantly less than 50% that produced in the control (●, black line). With C4/C12-HSL complementation, the elastase productions with 150 μM of DKST-1 (●, red line) and DKST-2 (●, green line) were restored in 4 h and completely diminished 8 h post complementation. C4/C12-HSL complementation was not effective in elastase production in Δ*dksA1* mutant (■, black line and □, black line).

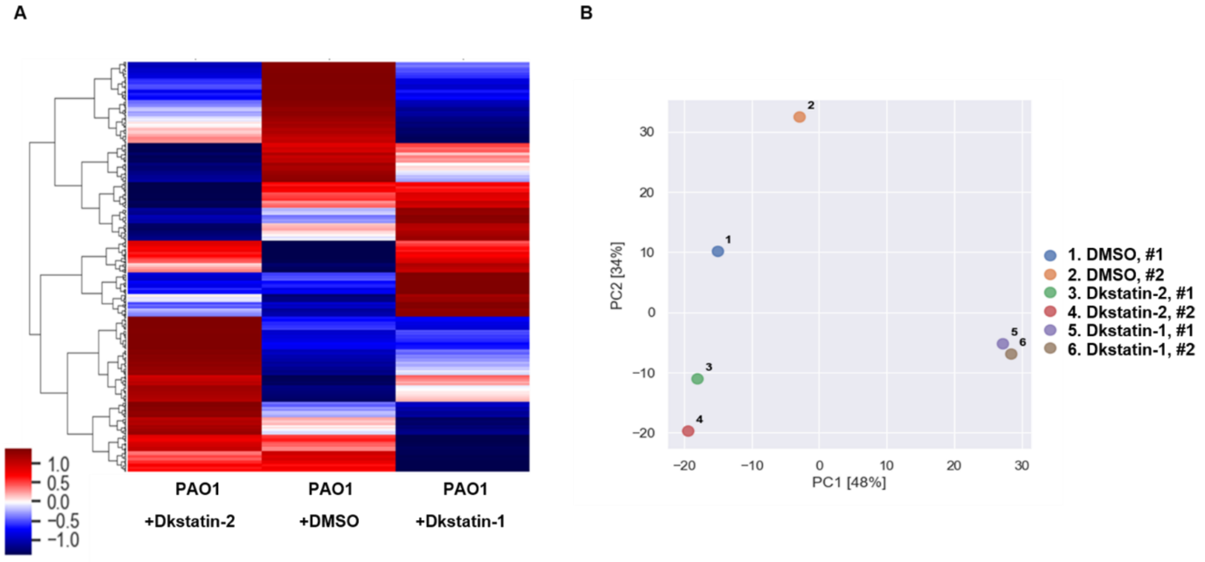

Fig. S4. Transcriptome analysis produced in PAO1 and PAO1 treated with Dkstatin-1 or Dkstatin-2. (**A**) Transcriptome heat-map produced from biologically duplicated RPKM values of PAO1 and Dkstatin-1 or -2 treated PAO1. DMSO was used as a solvent control for Dkstatin-1 and Dkstatin-2 treatment. The Z-score ranged from -1.0 to 1.0 and is represented as a color coded index. (**B**) PCA plot representing the similarity of transcripts in PAO1 treated with DMSO (#1 and #2) and with Dkstatin-1 (#5 and #6) or Dkstatin-2 (#3 and #4). Each similarity was significantly distinguishable. Total transcripts were harvested as biological duplicates.

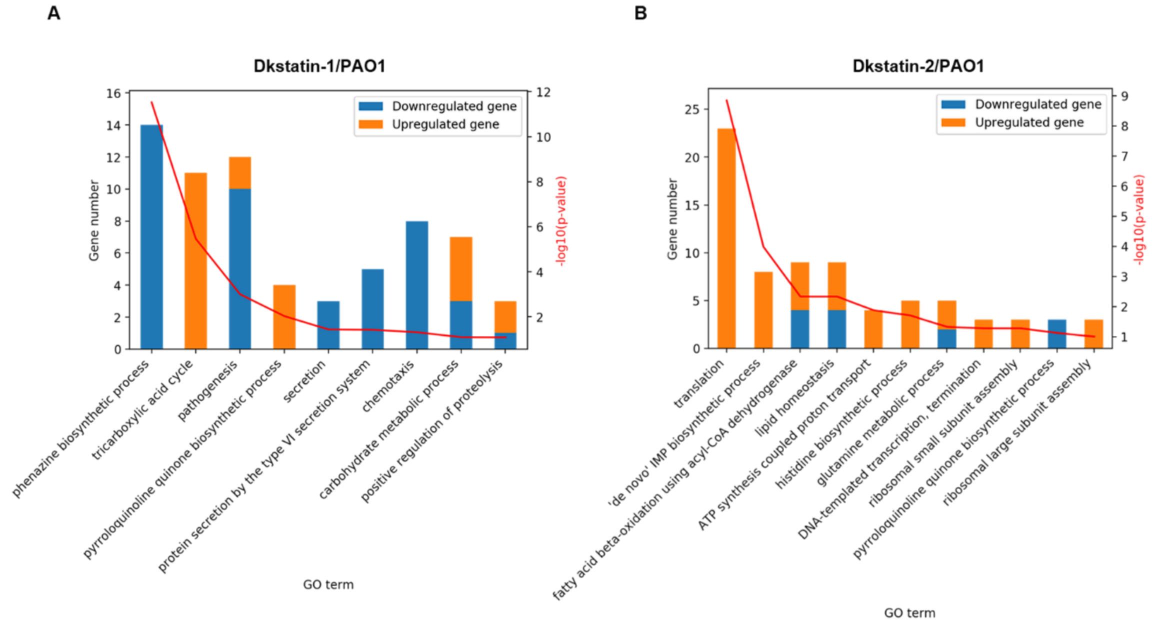

Fig. S5. Categorization of differentially expressed genes based on gene ontology (GO) terms. (**A**) GO terms in Dkstatin-1 treated group. Most genes included in GO terms (such as phenazines biosynthesis process, pathogenesis, secretion and chemotaxis) were down-regulated by Dkstatin-1 treatment. (**B**) Presented GO terms in Dkstatin-2 treated group. 11 GO terms were retrieved in the Dkstatin-2 treated group. Genes participating in GO terms such as translation, ribosomal assembly and energy metabolism were up-regulated by Dkstatin-2 treatment.
